# Apathy in patients with schizophrenia: Treatment perspectives

**DOI:** 10.1192/j.eurpsy.2021.161

**Published:** 2021-08-13

**Authors:** S. Kaiser

**Affiliations:** Adult Psychiatry Division, Department Of Psychiatry, University of Geneva Hospitals, Geneva, Switzerland

**Keywords:** schizophrénia, negative symptoms, apathy, Treatment

## Abstract

Apathy occurs in many neuropsychiatric disorders and is a central negative symptom of schizophrenia. Apathy has severe functional consequences for patients with schizophrenia and the development of evidence-based treatments is a major challenge. There is now increasing evidence that dysfunctions in reward processing underly apathy, in particular regarding reward anticipation, cost-benefit computation and reward learning. In addition, metacognitive processes such as defeatist performance beliefs modulate reward processing. Psychological interventions for negative symptoms target these processes. While the evidence for cognitive-behavioral therapy for negative symptoms remains limited, recent findings suggest that specifically targeting reward-related dysfunctions may improve efficacy of these interventions. On the neurobiological level, there is now considerable evidence that a dysregulation of the dopaminergic reward system is related to reward processing dysfunctions. Regarding pharmacological treatment approaches, psychostimulants have successfully been used for apathy in dementia to target the reward system. Pro-dopaminergic drugs to target apathy in schizophrenia seem to be safer than anticipated, but their efficacy remains to be established. At the current state of knowledge, there is no evidence-based treatment that specifically targets apathy in patients with schizophrenia today. However, there are encouraging results from research inspired by basic research in neuroscience and clinical research in patients with other neuropsychiatric disorders.

**Disclosure:**

SK has received royalties from Schuhfried (Austria) for cognitive test and training software.

